# Effects of Sodium Hypochlorite Concentration and Final Irrigation Protocol on Residual Filling Material in Retreated Oval Root Canals Obturated with a Calcium Silicate-Based Sealer: A Scanning Electron Microscopy Study

**DOI:** 10.3390/medicina62081593

**Published:** 2026-08-19

**Authors:** Damlanur Çetintaş, Melis Oya Ateş

**Affiliations:** Department of Endodontics, Faculty of Dentistry, Bolu Abant Izzet Baysal University, Bolu 14030, Türkiye; damlanur.bonceoglu@ibu.edu.tr

**Keywords:** root canal retreatment, calcium silicate-based sealer, final irrigation protocol, scanning electron microscopy, sodium hypochlorite

## Abstract

*Background and Objectives:* Residual filling material may persist on the walls of oval root canals after mechanical retreatment, and the influence of sodium hypochlorite (NaOCl) concentration and supplementary irrigation procedures on its removal remains unclear. This laboratory study used scanning electron microscopy (SEM) to evaluate the effects of three NaOCl concentrations and five final irrigation protocols on residual filling material in retreated oval canals of mandibular premolars. The protocols tested were conventional needle irrigation (CNI), EDDY, passive ultrasonic irrigation (PUI), XP-endo Finisher R (XPFR), and Shock Wave Enhanced Emission Photoacoustic Streaming (SWEEPS). *Materials and Methods:* One hundred twenty extracted mandibular premolars with a single root and an oval canal were instrumented with Reciproc R25 and filled with CeraSeal and a matching gutta-percha cone using the single-cone technique. Root canal retreatment was performed using ProTaper Universal Retreatment instruments followed by Reciproc R40. The teeth were then distributed among 15 experimental subgroups according to the final irrigation protocol and NaOCl concentration used: 2.5%, 5.25%, or 8.25% (*n* = 8). After irrigation, each root was divided longitudinally, and one undamaged canal half was randomly chosen for SEM evaluation. Residual filling material was scored in the coronal, middle, and apical regions using an ordinal scale ranging from 1 to 5. Examiner agreement was determined with quadratically weighted Cohen’s kappa, while treatment effects were examined using a cumulative link mixed model (CLMM). *Results:* Residual filling material scores differed significantly according to the final irrigation protocol (*p* < 0.001) and root canal region (*p* = 0.007), but not according to NaOCl concentration (*p* = 0.236). The interaction between protocol and canal region was also significant (*p* = 0.036). In the overall comparisons, CNI produced the highest scores, whereas SWEEPS produced the lowest, and EDDY, PUI, and XPFR did not differ significantly from one another. Apical scores were higher than coronal and middle scores in the EDDY, PUI, and SWEEPS protocols, while XPFR showed no significant variation among canal regions. *Conclusions:* Residual filling material scores after retreatment were influenced by the final irrigation protocol and root canal region, whereas NaOCl concentration had no significant main effect. SWEEPS resulted in the lowest overall residual filling material scores and CNI in the highest, while EDDY, PUI, and XPFR showed comparable overall performance, although protocol performance varied across root canal regions.

## 1. Introduction

Persistent or secondary intraradicular infection is one of the main reasons for post-treatment apical periodontitis [1]. When nonsurgical retreatment is required, the previous root canal filling must be removed to regain access to the canal system and permit further instrumentation, irrigation, disinfection, and obturation [2]. Material remaining on the dentinal walls may interfere with these procedures by preventing irrigants and medicaments from contacting previously covered surfaces and anatomical irregularities [3]. This challenge is particularly relevant in oval canals, where conventional instruments tend to prepare the central portion of the canal while leaving buccolingual recesses and other extensions relatively untouched [4].

Calcium silicate-based sealers (CSBSs) have become increasingly common in endodontic practice because of their biocompatibility, hydrophilic behavior, and bioactive properties [5]. However, mineral deposition associated with their hydration reaction, close adaptation to dentin, and penetration into dentinal tubules may complicate their removal during retreatment [6]. Previous studies have shown that remnants of CSBSs may persist on root canal walls after mechanical retreatment, particularly in anatomically complex or oval canals [7,8]. CeraSeal is a premixed CSBS commonly used with the single-cone technique [8]. Nevertheless, it remains unclear whether NaOCl concentration and supplementary final irrigation protocols influence the amount of residual filling material after retreatment of canals obturated with CeraSeal.

Several supplementary approaches have been proposed to improve canal cleanliness after mechanical retreatment [9]. Conventional needle irrigation (CNI) provides a clinically relevant reference method, but irrigant exchange beyond the needle outlet is limited, particularly in the apical third [10]. Its effectiveness may also be reduced by vapor lock in a closed-end canal system [11]. EDDY uses a flexible polyamide tip to generate sonic oscillation and hydrodynamic movement within the irrigant [12], whereas passive ultrasonic irrigation (PUI) relies mainly on acoustic streaming generated by an ultrasonically activated tip [13]. XP-endo Finisher R (XPFR) is manufactured from a temperature-responsive alloy that expands within the canal and can mechanically contact recesses that conventional retreatment instruments may not reach [14,15]. SWEEPS uses Er:YAG laser energy to enhance the pressure waves produced by laser-induced cavitation bubbles, thereby increasing irrigant movement throughout the root canal system [16,17].

Previous investigations have shown that supplementary cleaning procedures can reduce the amount of filling material left after retreatment, although complete removal remains uncommon [18,19]. EDDY and PUI have generally provided cleaner canal surfaces than CNI in experimental models, but their performance appears to depend on canal anatomy and the root level examined [20,21]. Studies comparing XPFR with PUI have likewise reported findings that vary according to the sealer, irrigation procedure, and canal region [18,22]. SWEEPS has been associated with lower amounts of CSBS remnants than CNI or PUI under certain laboratory conditions [23]. Nevertheless, no supplementary procedure has consistently produced completely clean canal walls, and direct comparisons among sonic, ultrasonic, mechanical, and laser-assisted protocols remain limited, particularly when NaOCl concentration and root canal region are considered simultaneously [17,20].

NaOCl remains the principal endodontic irrigant because of its antimicrobial activity and ability to dissolve organic tissue [24]. Its action is influenced not only by concentration but also by contact time, temperature, irrigant renewal, and activation [25]. Increasing NaOCl concentration generally enhances its organic tissue-dissolving capacity; however, higher concentrations, including 8.25%, may also adversely affect dentin properties [25,26]. In the present study, 8.25% NaOCl was included as a high-concentration experimental condition to extend the concentration range and to examine whether increasing NaOCl concentration could facilitate residual filling material removal after mechanical retreatment. Its inclusion was intended to evaluate an upper experimental concentration under controlled laboratory conditions rather than to represent routine clinical practice. Importantly, the material evaluated in the present study was not organic tissue but residual set CSBS. Because set CSBSs are predominantly inorganic and may interact closely with root dentin [5,27], the concentration-dependent organic tissue-dissolving action of NaOCl should be distinguished from its potential role in facilitating the removal of set sealer material. More specifically, the individual and interaction effects of NaOCl concentration, final irrigation protocol, and root canal region on residual filling material have not been sufficiently clarified. The principal novelty of the present study lies in the simultaneous factorial evaluation of five final irrigation protocols, three NaOCl concentrations, and three root canal regions within the same standardized oval-canal retreatment model, enabling these factors to be assessed within a common experimental framework.

Accordingly, this study used SEM to assess residual root canal filling material in the coronal, middle, and apical regions of oval mandibular premolars after standardized mechanical retreatment. Three NaOCl concentrations, 2.5%, 5.25%, and 8.25%, were tested with five final irrigation protocols: CNI, EDDY, PUI, XPFR, and SWEEPS. The study specifically examined whether increasing NaOCl concentration would provide any additional benefit in facilitating residual filling material removal and whether this effect would depend on the final irrigation protocol or root canal region. The null hypothesis was that residual filling material scores would not differ according to final irrigation protocol, NaOCl concentration, or root canal region and that no significant interactions would occur among these factors.

## 2. Materials and Methods

### 2.1. Ethical Approval

The experimental use of extracted human teeth was conducted in accordance with the Declaration of Helsinki and its subsequent amendments. Ethical clearance was granted by the Non-Interventional Clinical Research Ethics Committee of Bolu Abant Izzet Baysal University on 22 July 2025 (decision no. 2025/303). Before the teeth were collected, written consent permitting their use for research was obtained from the patients.

### 2.2. Sample Size Calculation

Sample size was estimated before the experiment using a simulation-based power analysis conducted in R statistical software, version 4.6.0, with the lme4 and simr packages. The simulated design reproduced the planned 5 × 3 factorial structure, comprising five irrigation protocols and three NaOCl concentrations, with three regional observations nested within each tooth. A tooth-level random intercept was included to account for the correlation among repeated regional observations within the same tooth.

As the intended outcome variable was ordinal, the calculation was approximated through a linear mixed-effects model (LMM). The power analysis was specifically designed to assess the three-way interaction among irrigation protocol, NaOCl concentration, and region. The anticipated effect size was specified as f^2^ = 0.35, based on previously published SEM-based ordinal scoring data from a root canal retreatment study [28]. The residual standard deviation was set to 1.0 (residual variance = 1.0), and the standard deviation of the tooth-level random intercept was set to 0.50 (random-intercept variance = 0.25). The three-way interaction was evaluated using a likelihood-ratio test, with α = 0.05 and a target power of 80%.

The simulation indicated that approximately 107 teeth were required under the specified assumptions. To ensure equal allocation across the 15 experimental subgroups and to allow for potential specimen loss, the final sample was increased to 120 teeth, corresponding to eight teeth per subgroup. Three regional SEM scores were recorded per specimen, resulting in 360 observations. The tooth was considered the independent experimental unit.

### 2.3. Specimen Selection and Standardization

The sample consisted of 120 extracted permanent mandibular premolars with one root and one oval-shaped canal. The teeth had been removed for orthodontic or periodontal indications that were unrelated to the study. Buccolingual and mesiodistal digital radiographs were taken to verify the presence of a single canal and to assess canal configuration. Canal morphology was considered oval when the buccolingual diameter was at least twice the mesiodistal diameter at a level 5 mm coronal to the root apex [29]. Canal curvature was calculated according to Schneider’s method, and only teeth with a curvature below 5° were accepted [30].

Each specimen was inspected at ×20 magnification with a dental operating microscope (DOM; Zumax Medical Co., Ltd., Suzhou, China). Eligibility criteria included a completely formed apex and the absence of caries, restorations, previous endodontic treatment, additional canals, cracks, fractures, discoloration, calcification, or internal or external resorption. Teeth with an apical foramen larger than a size 15 K-file were not included. Any specimen that failed to satisfy the selection criteria was replaced.

A digital caliper (BTS 12043; BTS, Istanbul, Türkiye) was used to measure total tooth length as well as crown and root lengths. The accepted dimensions were 20 ± 2 mm for total length, 5 ± 1 mm for crown length, and 15 ± 1 mm for root length. The selected root length range was consistent with that used in an earlier laboratory investigation involving mandibular premolars with oval canals [31].

Residual periodontal tissue was removed from the external root surface with a periodontal curette while the specimens were viewed under DOM magnification. The teeth were disinfected by immersion in 0.1% thymol for 48 h and were subsequently kept in distilled water at 4 °C until use. The storage medium was renewed regularly, and no specimen was stored for longer than 8 weeks [32]. All subsequent preparation, obturation, retreatment, and final irrigation procedures were completed under DOM magnification by a single postgraduate trainee in the final year of specialty training in endodontics (D.Ç.), under the supervision of an experienced endodontist (M.O.A.).

### 2.4. Root Canal Preparation

Access to the canal was obtained under water cooling with a round diamond bur (#801-016; Frank Dental, Gmund am Tegernsee, Germany). A size 15 K-file (Dentsply Maillefer, Ballaigues, Switzerland) was advanced through the canal to verify patency. When its tip became visible at the apical foramen, this length was recorded, and the working length was established 1 mm shorter [20]. A closed-end canal model was established by embedding each root in C-silicone impression material (Zetaplus; Zhermack, Badia Polesine, Italy), thereby limiting irrigant escape through the apical foramen [33].

Canal preparation was completed to the predetermined working length with Reciproc R25 (25/0.08; VDW, Munich, Germany) in the “Reciproc ALL” mode of an X-Smart Plus motor (Dentsply Maillefer, Ballaigues, Switzerland). Each instrument was limited to one specimen. The instrument was advanced with three gentle pecking movements per cycle, removed from the canal, and wiped with sterile gauze moistened with 70% alcohol. After each cycle, 2.5 mL of 2.5% NaOCl was delivered through a 30 G side-vented needle (Endo-Top; Cerkamed, Stalowa Wola, Poland). These steps were repeated until the instrument reached the working length.

All NaOCl solutions used during the study were prepared immediately before use from a reagent-grade stock solution containing 10–15% available chlorine (Sigma-Aldrich, St. Louis, MO, USA). Before dilution, the actual available chlorine content of the stock solution was determined by iodometric titration. Distilled water was then used to prepare the required concentrations of 2.5%, 5.25%, and 8.25%.

On completion of canal preparation, the canals received 5 mL of 2.5% NaOCl, followed by 5 mL of 17% ethylenediaminetetraacetic acid (EDTA; CanalPro EDTA, Coltene, Altstätten, Switzerland) and 5 mL of distilled water. The canals were then dried with sterile paper points while maintaining slight residual moisture.

### 2.5. Root Canal Obturation

The canals were filled by the single-cone technique using Reciproc R25 gutta-percha cones (25/0.08; VDW, Munich, Germany) and CeraSeal (Meta Biomed Co., Ltd., Cheongju, Republic of Korea).

After tug-back had been confirmed, CeraSeal was injected until it became visible at the canal orifice. The R25 master cone was coated with a thin layer of sealer and placed at the working length. Excess gutta-percha extending into the access cavity was severed at the canal orifice with a heated instrument. Residual sealer was removed from the access cavity using alcohol-moistened cotton pellets, after which the access cavity was sealed with a temporary restorative material (Cavit-G; 3M ESPE, Seefeld, Germany).

The obturation quality was checked on digital radiographs obtained in both buccolingual and mesiodistal projections. Teeth displaying radiographic voids or fillings that did not adequately reach the working length were excluded and replaced. To permit complete setting of the sealer, the specimens were maintained for 3 weeks at 37 °C and 100% humidity.

### 2.6. Root Canal Retreatment

Removal of the root canal filling began with ProTaper Universal Retreatment D1 (30/0.09), D2 (25/0.08), and D3 (20/0.07) instruments used in sequence. Canal enlargement was subsequently continued to the working length with Reciproc R40 (40/0.06; VDW, Munich, Germany).

During removal of the filling material, the canal was irrigated with 2.5 mL of 2.5% NaOCl after each instrument change. Mechanical retreatment was considered complete when the R40 instrument had reached the working length, no additional filling material was visible on the instrument flutes, and radiographs obtained in the buccolingual and mesiodistal directions showed no radiographically detectable filling remnants [34].

**Figure 1:**
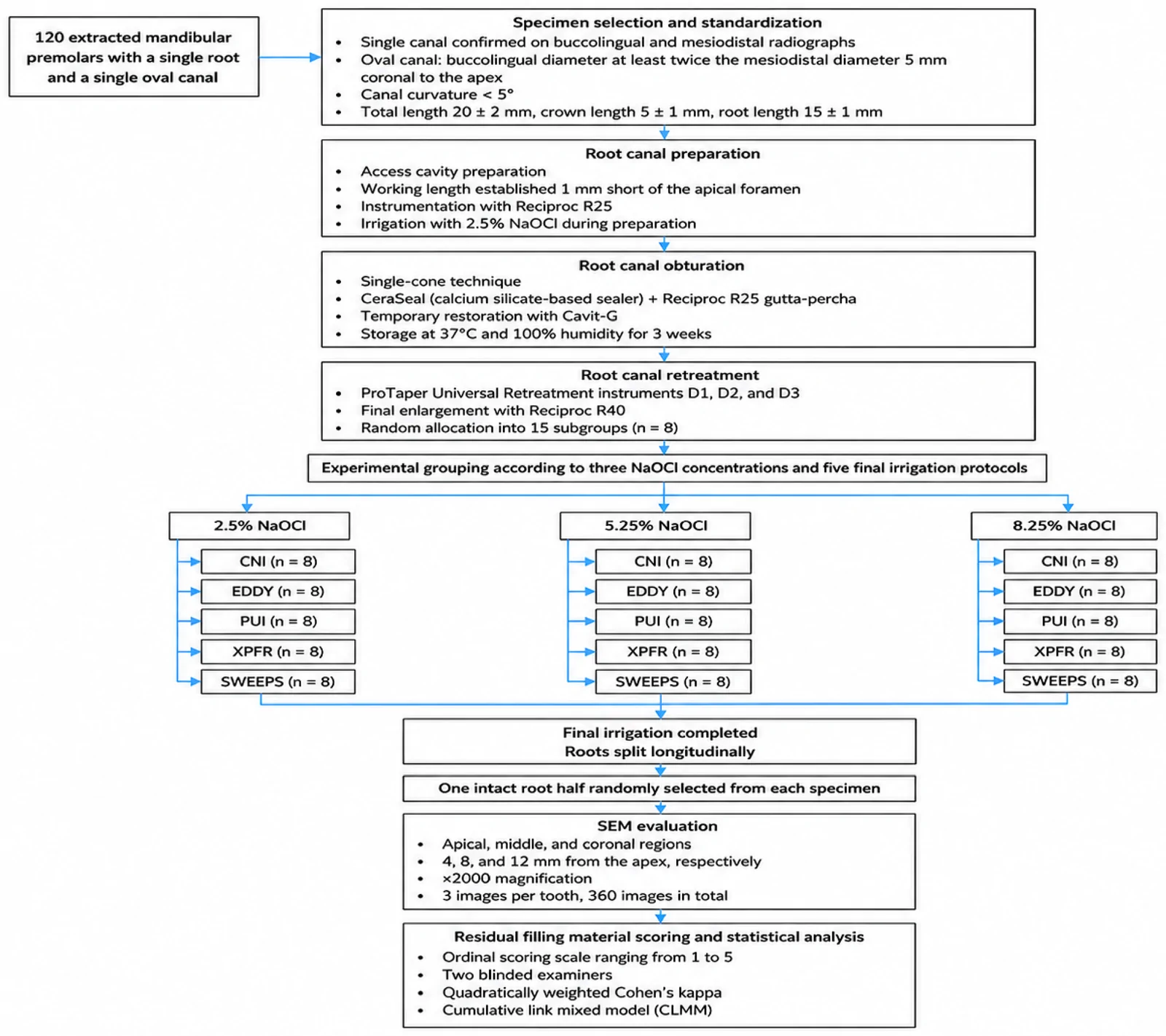
Flowchart of specimen selection, root canal preparation and obturation, retreatment, experimental grouping, final irrigation protocols, SEM evaluation, and statistical analysis.

After completion of the mechanical retreatment procedures, the teeth were numbered consecutively from 1 to 120. Random allocation was carried out with a computer-generated sequence obtained from Random.org. Equal numbers of specimens were assigned to 15 subgroups according to the combination of five final irrigation protocols and three NaOCl concentrations: 2.5%, 5.25%, and 8.25%. Each subgroup contained eight teeth. The experimental sequence is summarized in Figure 1.

### 2.7. Final Irrigation Protocols

The irrigants were administered in the same order in every group. The assigned NaOCl solution was used first for 1 min, followed by 17% EDTA for 1 min. A second 1 min NaOCl stage was then completed, and distilled water was used as the final rinse. Each stage involved 5 mL of solution.

For the EDDY, PUI, XPFR, and SWEEPS protocols, each 5 mL NaOCl stage was separated into two 30 s activation cycles. A fresh 2.5 mL aliquot was introduced immediately before each cycle. Thus, contact times and irrigant volumes were kept identical across the experimental groups; while NaOCl concentration and final irrigation protocol constituted the experimental factors.

#### 2.7.1. Conventional Needle Irrigation

CNI was used as the non-activated reference protocol. Irrigants were delivered with a 30 G side-vented needle positioned 1 mm short of the working length. The needle remained loose within the prepared canal and was moved gently through an apical–coronal range of approximately 3–4 mm.

The sequence comprised 5 mL of the assigned NaOCl concentration for 1 min, 5 mL of 17% EDTA for 1 min, a second 5 mL NaOCl application for 1 min, and a final 5 mL of rinse with distilled water.

#### 2.7.2. EDDY Sonic Activation

Sonic activation was carried out with an EDDY polyamide tip (25/0.04; VDW) attached to a Micron TA-200 Air Scaler (TA-200-S2H; Micron, Tokyo, Japan) operating at 6000 Hz. The tip was placed 1 mm short of the working length and kept free of binding against the canal walls. During activation, it was moved gently in an apical–coronal direction over a distance of 3–4 mm.

Each 5 mL NaOCl stage consisted of two cycles. Before each cycle, 2.5 mL of fresh solution was delivered with the 30 G side-vented needle, followed by 30 s of EDDY activation. An amount of 5 mL of 17% EDTA was activated sonically for 1 min using the same approach. After the second NaOCl stage, the canal was rinsed with 5 mL of distilled water [21].

#### 2.7.3. Passive Ultrasonic Irrigation

PUI was performed with an Ultra X ultrasonic unit and a size 20/0.02 ultrasonic tip (Eighteeth, Changzhou, China). The tip was positioned 1 mm short of the working length and operated at 45 kHz with the power set to its maximum level. It was allowed to oscillate freely without contacting the canal walls.

Each NaOCl stage was divided into two 30 s cycles, with 2.5 mL of newly delivered irrigant used for each cycle. The 5 mL EDTA stage was ultrasonically activated for 1 min. The same procedure was repeated for the second NaOCl application, followed by a final rinse with 5 mL of distilled water [20,21].

#### 2.7.4. XP-Endo Finisher R Protocol

The XP-endo Finisher R instrument (XPFR; FKG Dentaire, La Chaux-de-Fonds, Switzerland) was operated using an X-Smart Plus endodontic motor (Dentsply Maillefer, Ballaigues, Switzerland) at 1000 rpm and 1 N·cm. To promote the instrument’s temperature-dependent phase transformation, specimens allocated to this protocol were kept in a 37 °C water bath throughout instrument use [35]. A new XPFR instrument was assigned to each tooth.

After introducing 2.5 mL of NaOCl, the instrument was inserted to 1 mm short of the working length and moved with slow longitudinal strokes of 7–8 mm for 30 s [36]. The procedure was repeated using a second 2.5 mL volume of fresh NaOCl, giving a total NaOCl volume of 5 mL and an overall activation time of 1 min. The same instrument was subsequently used to activate 5 mL of 17% EDTA for 1 min. The NaOCl stage consisting of two cycles was then repeated, and the canal received a final 5 mL of distilled water rinse [21].

#### 2.7.5. SWEEPS Laser-Activated Irrigation

SWEEPS activation was performed with an Er:YAG laser system (LightWalker AT; Fotona, Ljubljana, Slovenia) fitted with a radial SWEEPS 600 fiber tip. The tip was held at the canal orifice and was not advanced into the root canal. AutoSWEEPS mode was selected with the following settings: 20 mJ per pulse, 15 Hz, and 0.60 W. Both air and water spray were switched off during laser activation [17,37].

For each cycle, 2.5 mL of NaOCl was delivered through the 30 G side-vented needle and activated for 30 s. Two cycles were completed, providing a total NaOCl volume of 5 mL and an overall activation time of 1 min. The subsequent 5 mL of 17% EDTA was laser activated for 1 min. The NaOCl stage was then repeated, followed by a final rinse with 5 mL of distilled water.

After the completion of all final irrigation procedures, the canals were dried with sterile R40 paper points (VDW).

### 2.8. Specimen Preparation for Scanning Electron Microscopy

A diamond disc was used to create longitudinal grooves on the mesial and distal external root surfaces, parallel to the long axis of each tooth. Care was taken to avoid entering the canal space or disturbing material attached to the canal walls. The roots were then separated into two longitudinal halves with a chisel. Teeth in which splitting caused fracture or damage to the canal surface were excluded and replaced. When both halves remained intact, one was selected randomly for SEM evaluation.

Reference points were marked 4, 8, and 12 mm from the anatomical apex to represent the apical, middle, and coronal regions, respectively. The specimens were dehydrated through increasing concentrations of ethanol, dried, mounted on aluminum stubs, and sputter-coated with a 90 Å thick gold layer.

Examination was carried out with a scanning electron microscope (SU5000; Hitachi High-Technologies Corporation, Tokyo, Japan) at an accelerating voltage of 5 kV. At each predefined reference level (4, 8, and 12 mm from the anatomical apex), the imaging field was selected using the same standardized geometric procedure for all specimens. The ×2000 field was centered on the central portion of the exposed canal wall surface at the marked reference level, and field selection was not based on the presence or amount of residual filling material. One micrograph was obtained from each of the three marked regions. Accordingly, three images were recorded per tooth and 360 SEM images were produced in total.

### 2.9. Residual Material Scoring and Reliability Assessment

Before formal scoring, the examiners were calibrated using representative SEM reference images of exposed dentin, residual sealer, gutta-percha, and smear/dentinal debris obtained from specimens not included in the final analysis, and agreed on the morphological criteria used to distinguish these features. Residual filling material was identified by comparison with these reference images and by its morphological distinction from the surrounding exposed dentinal surface. The sealer and gutta-percha components were not quantified separately; instead, they were assessed collectively as residual filling material.

Before assessment, every SEM image was divided into 100 equal fields using Adobe Photoshop CS4 (Adobe Systems Inc., San Jose, CA, USA). The proportion of the evaluated canal wall occupied by residual filling material was classified according to a previously described five-point ordinal scale [20] with the terminology adapted to residual filling material:

Score 1: Residual filling material covers less than 25% of the evaluated root canal wall surface.

Score 2: Residual filling material covers 25% to less than 50% of the evaluated root canal wall surface.

Score 3: Residual filling material covers 50% to less than 75% of the evaluated root canal wall surface.

Score 4: Residual filling material covers 75% to less than 100% of the evaluated root canal wall surface and appears in large aggregated or scattered amounts.

Score 5: Residual filling material covers the entire evaluated root canal wall surface.

All 360 SEM images were independently scored by two calibrated examiners who were blinded to the experimental allocation. Interexaminer agreement was assessed by comparing their initial ratings across the complete image set. Images receiving different initial scores were subsequently reviewed jointly, and a final consensus score was established for use in the main statistical analyses. Intraexaminer agreement was evaluated using a stratified random subset of 90 images. Two images were randomly selected from each of the 45 strata formed by the 15 experimental subgroups and three canal regions. Following a washout period of three weeks, each examiner independently rescored the selected images without access to the previous ratings or experimental allocation. Interexaminer and intraexaminer agreement were evaluated using quadratically weighted Cohen’s kappa coefficients with 95% confidence intervals. Kappa values were interpreted according to Landis and Koch [38].

### 2.10. Statistical Analysis

Because the residual filling material scores were ordinal and three repeated regional measurements were obtained from each tooth, the effects of the final irrigation protocol, NaOCl concentration, and root canal region were analyzed using a cumulative link mixed model (CLMM). Final irrigation protocol, NaOCl concentration, and root canal region were included as fixed effects, together with all two-way interactions and the three-way interaction among these factors. Tooth identity was included as a random intercept to account for the within-specimen correlation among the apical, middle, and coronal observations.

Overall fixed effects and interactions in the CLMM were evaluated using chi-square tests. When a statistically significant interaction was detected, pairwise simple-effects comparisons were performed within the relevant levels of the interacting factors using estimated marginal means. For the significant interaction between final irrigation protocol and root canal region, final irrigation protocols were compared separately within each root canal region, and root canal regions were compared separately within each final irrigation protocol. In the absence of a significant interaction, pairwise comparisons were performed only for statistically significant main effects. Because neither the main effect of NaOCl concentration nor any interaction involving NaOCl concentration was statistically significant, no concentration-specific post hoc comparisons were performed. Overall model-based comparisons among final irrigation protocols, averaged over NaOCl concentrations and root canal regions, were also reported and were interpreted in conjunction with the region-specific comparisons because of the significant protocol-by-region interaction. The Holm adjustment was applied within each family of comparisons to control the family-wise type I error rate. Interexaminer and intraexaminer agreement were quantified using quadratically weighted Cohen’s kappa coefficients with 95% confidence intervals.

Descriptive data were reported as medians and interquartile ranges. Statistical significance was set at *p* < 0.05. All analyses were performed using R statistical software, version 4.6.0 (R Foundation for Statistical Computing, Vienna, Austria). CLMMs were fitted using the ordinal package, version 2025.12-29, and estimated marginal means and multiplicity-adjusted post hoc comparisons were obtained using the emmeans package, version 2.0.4.

## 3. Results

### 3.1. Examiner Agreement

Interexaminer agreement for the initial ratings of all 360 SEM images was almost perfect (κ = 0.961; 95% CI, 0.858–1.000; *p* < 0.001). Intraexaminer agreement was also almost perfect for both examiners. The quadratically weighted Cohen’s kappa coefficient was 0.995 for Examiner 1 (95% CI, 0.892–1.000; *p* < 0.001) and 0.992 for Examiner 2 (95% CI, 0.889–1.000; *p* < 0.001) (Table 1).

### 3.2. Effects of Final Irrigation Protocol, NaOCl Concentration, and Root Canal Region

According to the CLMM, residual filling material scores varied significantly with the final irrigation protocol (*p* < 0.001) and root canal region (*p* = 0.007). NaOCl concentration, however, did not have a significant main effect (*p* = 0.236).

Of the interaction terms examined, only the interaction between final irrigation protocol and root canal region was statistically significant (*p* = 0.036). No significant interaction was found between final irrigation protocol and NaOCl concentration (*p* = 0.834), between NaOCl concentration and root canal region (*p* = 0.939), or among all three factors (*p* = 0.976) (Table 2).

Because neither NaOCl concentration nor any interaction involving concentration reached statistical significance, the primary post hoc comparisons focused on the relationship between the final irrigation protocol and root canal region, with results averaged over the three NaOCl concentrations.

### 3.3. Comparisons by Final Irrigation Protocol and Root Canal Region

The regional pattern of residual filling material scores differed among the final irrigation protocols (Table 3). Within the EDDY protocol, the apical region had significantly higher scores than both the coronal (adjusted *p* < 0.001) and middle (adjusted *p* = 0.009) regions, whereas the coronal and middle regions did not differ significantly (adjusted *p* = 0.083). For CNI, scores were significantly higher in the middle than in the coronal region (adjusted *p* = 0.006), and in the apical than in the coronal region (adjusted *p* < 0.001); the middle and apical regions did not differ significantly (adjusted *p* = 0.351). For SWEEPS, the apical region had significantly higher scores than both the coronal (adjusted *p* = 0.015) and middle (adjusted *p* = 0.015) regions, while the coronal and middle regions did not differ significantly (adjusted *p* = 0.981). Similarly, for PUI, the apical region had significantly higher scores than both the coronal (adjusted *p* < 0.001) and middle (adjusted *p* < 0.001) regions, with no significant difference between the coronal and middle regions (adjusted *p* = 0.335). No significant regional differences were observed for XPFR (all adjusted *p* ≥ 0.05).

Pairwise comparisons among the five final irrigation protocols were performed separately within the coronal, middle, and apical regions (Table 4). In the coronal region, CNI had significantly higher residual filling material scores than EDDY, SWEEPS, PUI, and XPFR (all adjusted *p* < 0.001). SWEEPS had significantly lower scores than PUI and XPFR (both adjusted *p* < 0.001). In the middle region, CNI had significantly higher scores than all other protocols (all adjusted *p* < 0.001). SWEEPS had significantly lower scores than EDDY (adjusted *p* = 0.002), PUI (adjusted *p* = 0.003), and XPFR (adjusted *p* < 0.001). Similarly, in the apical region, CNI had significantly higher scores than all other protocols (all adjusted *p* < 0.001). EDDY had significantly higher scores than SWEEPS (adjusted *p* = 0.001) and XPFR (adjusted *p* = 0.018), but significantly lower scores than PUI (adjusted *p* = 0.028). SWEEPS had significantly lower scores than PUI (adjusted *p* < 0.001), and PUI had significantly higher scores than XPFR (adjusted *p* < 0.001).

**Figure 2:**
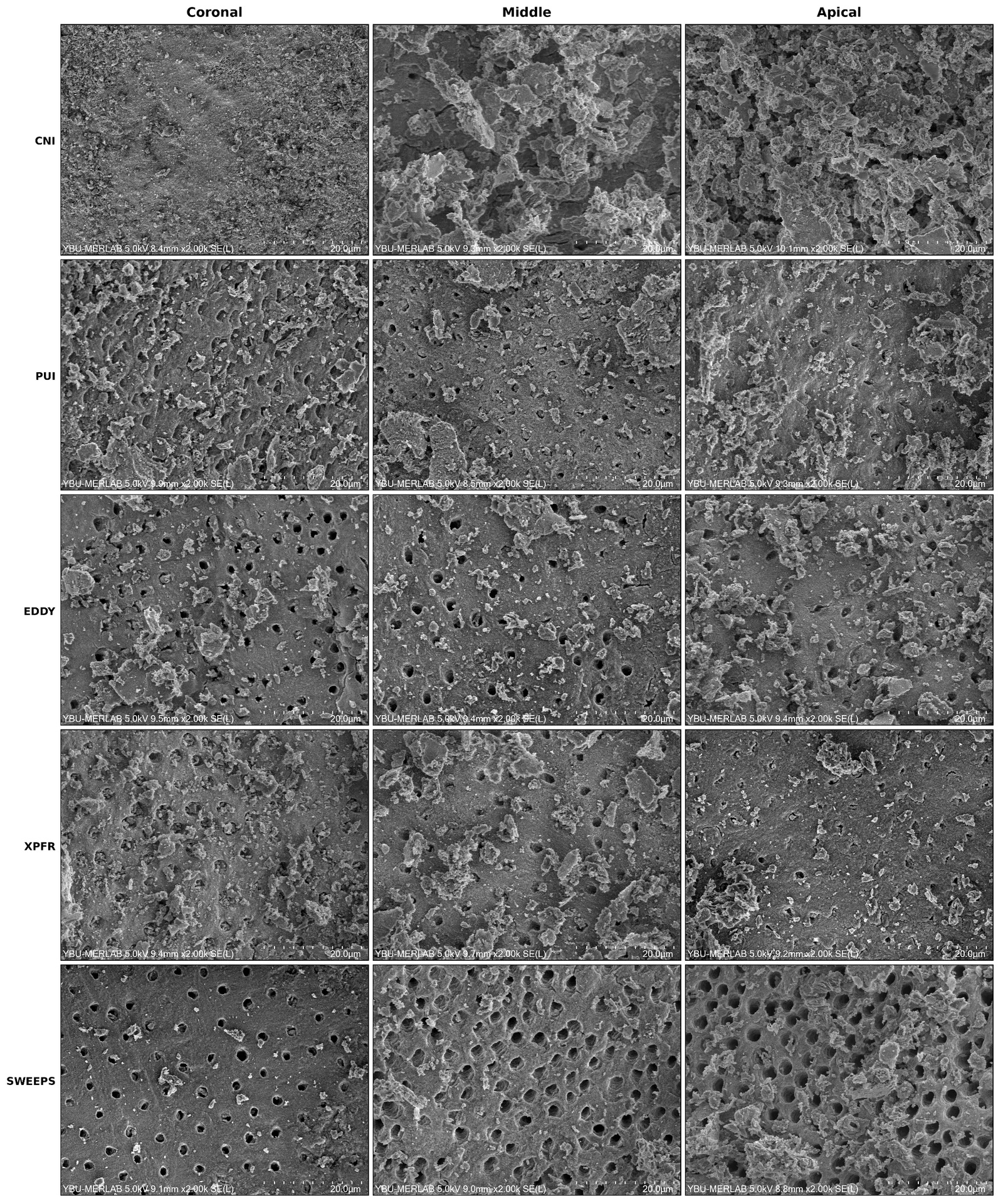
Representative SEM images (×2000) of residual root canal filling material in the coronal, middle, and apical regions following different final irrigation protocols. All images shown were obtained from the 5.25% NaOCl subgroups.

Figure 2 presents representative SEM micrographs of residual filling material in the coronal, middle, and apical regions after each final irrigation protocol. For consistency of visual presentation, all displayed micrographs were selected from the 5.25% NaOCl subgroups. The images are intended for illustrative purposes and not for comparisons among NaOCl concentrations.

### 3.4. Overall Comparisons Among Final Irrigation Protocols

**Figure 3:**
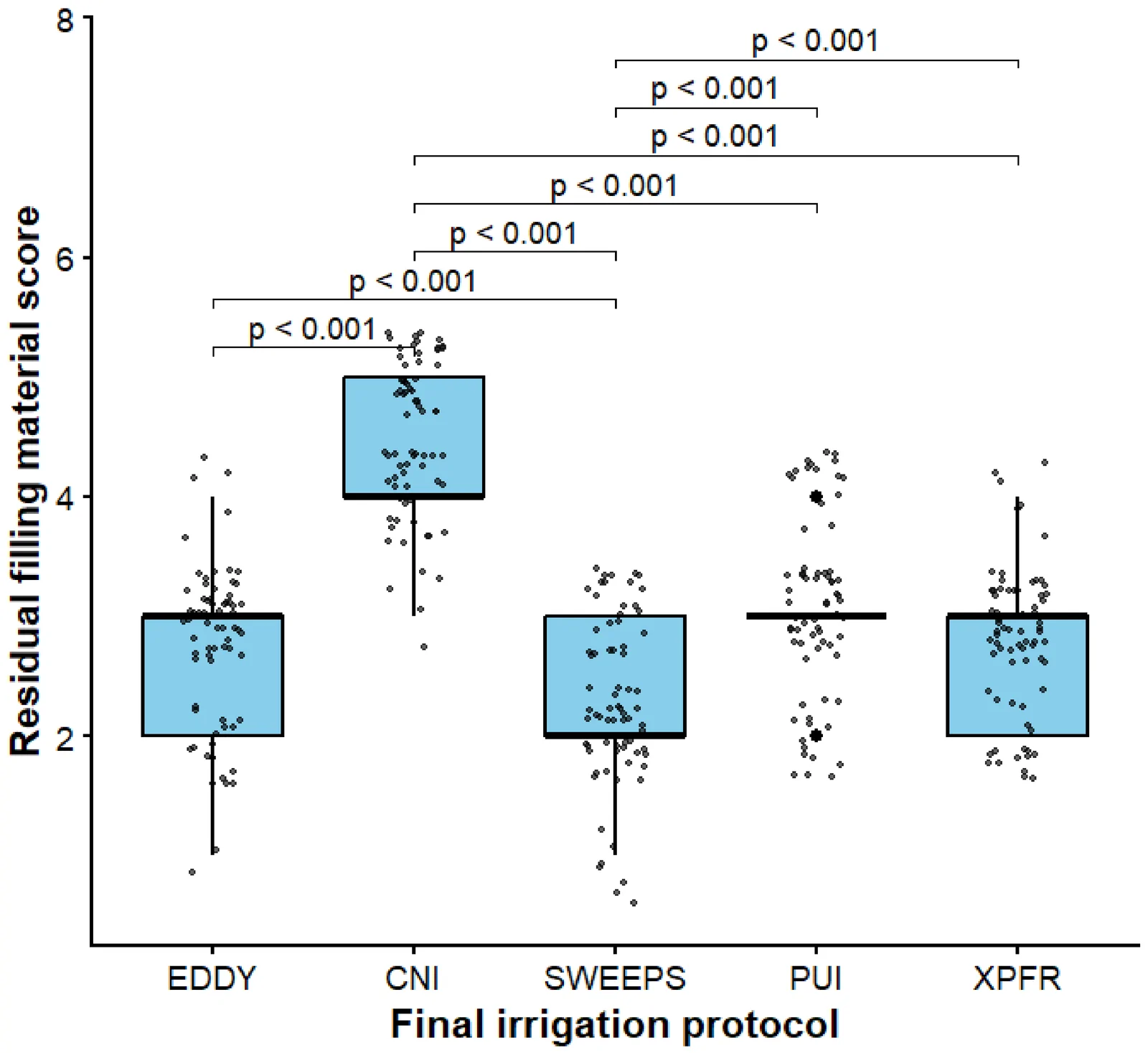
Distribution of residual filling material scores according to final irrigation protocol and results of model-based pairwise comparisons among protocols. Boxes represent the interquartile range, horizontal lines indicate the median, and individual points represent observed scores. Pairwise comparisons were based on estimated marginal means derived from the CLMM and averaged over the levels of NaOCl concentration and root canal region. The Holm method was used to adjust the *p* values.

When the comparisons were averaged over NaOCl concentrations and root canal regions, CNI produced higher residual filling material scores than EDDY, PUI, XPFR, and SWEEPS (all adjusted *p* < 0.001). SWEEPS, in turn, produced lower scores than EDDY, PUI, and XPFR (all adjusted *p* < 0.001). The overall scores of EDDY, PUI, and XPFR were not significantly different from one another (all adjusted *p* > 0.05) (Figure 3). Descriptive values and the full set of pairwise comparisons corresponding to Figure 3 are reported in Supplementary Table S1. These overall comparisons should be interpreted in conjunction with the region-specific comparisons presented in Table 4 because of the significant interaction between final irrigation protocol and root canal region.

### 3.5. Effect of NaOCl Concentration

Residual filling material scores did not differ significantly among the 2.5%, 5.25%, and 8.25% NaOCl concentrations when evaluated across the entire dataset. Likewise, none of the interactions involving NaOCl concentration was statistically significant. The descriptive findings according to final irrigation protocol, NaOCl concentration, and root canal region are provided in Supplementary Table S2.

## 4. Discussion

The findings indicate that the effectiveness of final irrigation after retreatment depended on both the irrigation protocol and root canal region, whereas NaOCl concentration had no significant main effect. The significant protocol-by-region interaction further indicates that protocol performance was not uniform throughout the canal. Accordingly, the null hypothesis was partially rejected.

All specimens underwent the same standardized mechanical retreatment sequence using ProTaper Universal Retreatment instruments followed by Reciproc R40 before allocation to the final irrigation protocols, thereby holding mechanical retreatment kinematics constant across the experimental groups. A recent systematic review found no clear evidence that continuous rotary instruments produce less apically extruded debris than reciprocating instruments during nonsurgical endodontic retreatment [39]. Although apical debris extrusion was not evaluated in the present study, the findings of that review suggest that retreatment kinematics alone may not be the principal determinant of apical extrusion and that instrument design, irrigation protocol, and experimental methodology should also be considered.

CNI was associated with the highest residual filling material scores, highlighting the limitations of needle irrigation after mechanical retreatment. With needle irrigation, irrigant delivery depends on the depth of needle insertion, solution renewal, and the flow generated around the needle outlet. Beyond the needle tip, fluid replacement becomes progressively more limited, especially in the apical part of the canal and in buccolingual extensions that remain untouched by instruments [10,40]. Supplementary protocols address these limitations through enhanced fluid movement, direct mechanical contact, or photoacoustic effects. Previous studies have likewise reported improved filling material removal with supplementary irrigation compared with CNI [20,21,41].

SWEEPS produced the lowest overall scores among the tested protocols. Cavitation generated by Er:YAG laser pulses may enhance irrigant movement beyond areas directly accessible to instruments, including oval recesses [16]. Angerame et al. [23] similarly reported less bioceramic filling material after SWEEPS than after ultrasonic activation. Laser-assisted irrigation has also been shown to reduce gutta-percha and tricalcium silicate-based sealer remnants [42]. Variations in sealer type, canal configuration, irrigant volume, activation time, laser parameters, tip placement, and assessment method may explain differences among published studies.

Although EDDY, PUI, and XPFR work through different principles, their overall scores were comparable. EDDY generates sonic movement, while PUI relies on ultrasonic oscillation and the resulting acoustic streaming. XPFR differs from both because it combines irrigant agitation with direct mechanical contact along irregular canal walls. After retreatment enlargement to R40, these different mechanisms may have produced a similar overall reduction in residual material. Comparable results for sonic and ultrasonic activation have been reported previously, despite marked differences in frequency and tip design [12,41]. Güngördü et al. [21] also found that EDDY, PUI, and XPFR improved filling material removal in oval mandibular premolars compared with needle irrigation, without identifying a consistently superior method among the three.

The significant protocol-by-region interaction suggests that protocol performance in residual filling material removal varied along the root canal. The apical region remained particularly challenging for several activation protocols, which may be related to reduced irrigant exchange, canal narrowing, anatomical irregularities, and vapor lock in a closed-end model [11]. Retreatment remnants may also be displaced and compacted apically [7,43], consistent with previous observations after sonic and ultrasonic irrigation [41,44].

XPFR showed a more uniform regional pattern than the other supplementary protocols. The temperature-responsive MaxWire alloy can undergo phase transformation and expand within the canal, while the instrument’s longitudinal motion may increase mechanical contact with buccolingual extensions that are difficult to reach with conventional retreatment instruments [22,45]. This adaptive behavior may help explain why a distinct apical increase in the scores was not observed in the XPFR group.

Across the range tested, no measurable additional benefit in residual filling material removal was detected with increasing NaOCl concentration from 2.5% to 8.25%. Although higher NaOCl concentrations are associated with greater antimicrobial and tissue-dissolving activity [46,47,48], the material evaluated here was a set CSBS rather than organic tissue. Once hardened, these sealers are predominantly inorganic and may establish both chemical and micromechanical interactions with root dentin. Their ability to penetrate dentinal tubules and promote mineral formation may also reduce their susceptibility to chemical removal [5,8].

Previous retreatment studies have shown that CSBS remnants may remain attached to canal walls or within dentinal tubules after mechanical enlargement and supplementary irrigation [20,22,42]. Such remnants, when present, could reduce the contact of irrigants or intracanal medicaments with previously covered dentin. From a clinical perspective, a supplementary cleaning protocol may improve filling material removal after mechanical retreatment of canals obturated with a CSBS. Although SWEEPS resulted in the lowest scores under the conditions of the present laboratory study, this finding does not establish superior antimicrobial efficacy, periapical healing, or long-term treatment success. These outcomes were not evaluated in the present study, and clinical recommendations regarding a particular irrigation protocol or NaOCl concentration should therefore remain cautious.

Several limitations need to be considered. The experiment involved only mandibular premolars with single oval canals and one CSBS. The results may consequently differ with other tooth types, canal configurations, sealers, or obturation procedures. Although predefined anatomical eligibility criteria were applied during specimen selection and specimens were randomly allocated to the experimental subgroups, individual baseline anatomical measurements were not retained in a form that allowed reliable retrospective comparison of root length, canal curvature, and buccolingual-to-mesiodistal diameter ratios among the 15 subgroups. Therefore, anatomical comparability among the randomized subgroups could not be directly demonstrated. Furthermore, canal morphology was assessed using two-dimensional buccolingual and mesiodistal radiographs, which do not provide a complete three-dimensional characterization of oval root canal anatomy. Micro-CT-based characterization and matching could have enabled more detailed assessment and balancing of canal volume, surface area, and morphological irregularities among the experimental groups. In addition, the initial volume and three-dimensional distribution of the obturation material were not quantified before retreatment. Therefore, despite the standardized obturation procedure, baseline equivalence in the amount and distribution of filling material among specimens and experimental groups could not be directly demonstrated. These factors should be considered when interpreting the findings.

The sample size calculation has limitations because an LMM was used for prospective planning, whereas the final analysis was performed using a CLMM. An LMM assumes a continuous response with normally distributed residuals and constant residual variance, whereas a CLMM models an ordinal outcome through cumulative probabilities and threshold parameters. Therefore, the estimated 80% power should be regarded as an approximate planning estimate rather than an exact estimate of the power of the final CLMM. In addition, the study was not necessarily equally powered for all main effects and interaction terms; in particular, smaller two-way and three-way interactions may have had lower statistical power. Consequently, nonsignificant interaction effects should not be interpreted as evidence that the corresponding interactions were definitively absent.

In addition, during the final irrigation procedures, only the XPFR specimens were maintained at 37 °C to promote the temperature-dependent phase transformation of the instrument, whereas the same thermal condition was not applied to the other irrigation protocols. Temperature therefore represents a potential confounding factor in comparisons involving XPFR because it may influence NaOCl behavior [25] as well as the temperature-dependent behavior of the XP-endo instrument [35]. Consequently, the observed XPFR results may reflect the combined effects of its mechanical action and the thermal condition under which it was used. Because temperature was not independently manipulated across protocols, its specific contribution cannot be separated from the protocol effect in the present experimental design. Future studies should standardize thermal conditions across protocols or investigate temperature as an independent experimental factor.

Although a standardized field-selection procedure was used, only one intact root half and a single ×2000 micrograph from each predefined canal level were examined. Therefore, each image represented only a limited portion of the canal-wall surface and may not fully reflect the spatial heterogeneity of residual filling material within the corresponding canal region. Dividing each micrograph into 100 fields standardized scoring within the captured image but did not increase the spatial coverage of SEM sampling. Because no energy-dispersive spectroscopy or other complementary compositional analysis was performed, definitive compositional identification of all surface deposits could not be established.

SEM allowed detailed examination of selected canal wall areas at high magnification; however, the SEM-based five-point scoring system provided an ordinal, surface-based assessment of residual filling material rather than a quantitative three-dimensional measurement of the remaining material. In addition, the lowest category of the ordinal scoring system included surface coverage ranging from complete absence to less than 25% residual filling material. Therefore, the scoring system did not allow a completely clean SEM field to be distinguished unequivocally from a field containing a small amount of residual material. Consequently, the presence or absence of residual filling material in individual images cannot be inferred solely from a score of 1. Accordingly, the findings reflect differences in these scores and cannot be interpreted as direct measurements of the volume or three-dimensional distribution of residual material. A quantitative three-dimensional method, such as micro-CT, could have provided complementary information regarding the volume and spatial distribution of the remaining filling material. Furthermore, the ordinal scoring method remained partly dependent on examiner judgment despite calibration and the high level of agreement obtained. The laboratory model was also unable to reproduce all clinical conditions, including periodontal tissue resistance, intracanal fluid behavior, limitations of clinical access, and patient-related factors. Future work should include different CSBSs and canal anatomies, compare a wider range of activation conditions and temperatures, and combine surface assessment with three-dimensional imaging and clinically relevant microbiological outcomes.

## 5. Conclusions

Under the conditions of this laboratory model, residual filling material scores varied according to the final irrigation protocol and root canal region, whereas NaOCl concentration had no significant main effect. CNI produced the highest scores, whereas SWEEPS produced the lowest. EDDY, PUI, and XPFR showed comparable overall performance, although protocol performance varied across root canal regions.

## Figures and Tables

**Table 1:** Quadratically weighted Cohen’s kappa coefficients for interexaminer and intraexaminer agreement in SEM residual filling material scoring.

Type of Agreement	Comparison	Images, *n*	Quadratically Weighted Cohen’s κ	95% CI	*p* Value
Interexaminer agreement	Examiner 1 vs. Examiner 2, initial ratings	360	0.961	0.858–1.000	<0.001
Intraexaminer agreement	Examiner 1, initial vs. repeat ratings	90	0.995	0.892–1.000	<0.001
Intraexaminer agreement	Examiner 2, initial vs. repeat ratings	90	0.992	0.889–1.000	<0.001

**Table 2:** Main and interaction effects of the final irrigation protocol, NaOCl concentration, and root canal region on residual filling material scores based on the cumulative link mixed model.

Effect	χ^2^	Degrees of Freedom	*p* Value
Final irrigation protocol	50.050	4	**<0.001**
NaOCl concentration	2.891	2	0.236
Root canal region	9.852	2	**0.007**
Final irrigation protocol × NaOCl concentration	4.252	8	0.834
Final irrigation protocol × root canal region	16.472	8	**0.036**
NaOCl concentration × root canal region	0.796	4	0.939
Final irrigation protocol × NaOCl concentration × root canal region	6.855	16	0.976

Bold *p* values indicate statistical significance at α = 0.05.

**Table 3:** Distribution of residual filling material scores in the coronal, middle, and apical regions according to final irrigation protocol and results of within-protocol regional comparisons. Descriptive values were pooled across the three NaOCl concentrations, and model-based comparisons were averaged over these concentrations.

	Root Canal Regions			Adjusted *p* Values		
Final İrrigation Protocol	Coronal	Middle	Apical	Coronal vs. Middle	Coronal vs. Apical	Middle vs. Apical
EDDY	2.5 (2–3)	3 (2–3)	3 (3–3)	0.083	**<0.001**	**0.009**
CNI	4 (4–4)	5 (4–5)	5 (4–5)	**0.006**	**<0.001**	0.351
SWEEPS	2 (2–2)	2 (2–3)	3 (2–3)	0.981	**0.015**	**0.015**
PUI	3 (2.75–3)	3 (2–3)	3.5 (3–4)	0.335	**<0.001**	**<0.001**
XPFR	3 (2.75–3)	3 (2.75–3)	3 (2–3)	0.447	0.556	0.220

**Note:** Data are presented as median (interquartile range) and were pooled across the three NaOCl concentrations. Within-protocol regional comparisons were evaluated using estimated marginal means derived from the cumulative link mixed model. Pairwise comparisons were adjusted using the Holm method. Bold *p* values indicate statistical significance at α = 0.05.

**Table 4:** Pairwise comparisons of final irrigation protocols within each root canal region.

	Root Canal Regions		
Comparison	Coronal	Middle	Apical
EDDY–CNI	**<0.001**	**<0.001**	**<0.001**
EDDY–SWEEPS	0.120	**0.002**	**0.001**
EDDY–PUI	0.085	0.879	**0.028**
EDDY–XPFR	0.120	0.260	**0.018**
CNI–SWEEPS	**<0.001**	**<0.001**	**<0.001**
CNI–PUI	**<0.001**	**<0.001**	**<0.001**
CNI–XPFR	**<0.001**	**<0.001**	**<0.001**
SWEEPS–PUI	**<0.001**	**0.003**	**<0.001**
SWEEPS–XPFR	**<0.001**	**<0.001**	0.318
PUI–XPFR	0.787	0.260	**<0.001**

**Note:** Within-region comparisons among final irrigation protocols were evaluated using estimated marginal means derived from the cumulative link mixed model and averaged over the three NaOCl concentrations. Pairwise *p* values were adjusted using the Holm method. Bold *p* values indicate statistical significance at α = 0.05.

## Data Availability

The data supporting the findings of this study are provided within the article. Further information may be obtained from the corresponding author upon reasonable request.

## Supplementary Materials

The following supporting information can be downloaded at https://www.mdpi.com/article/10.3390/medicina62081593/s1. Table S1: Distribution of residual filling material scores according to final irrigation protocols and results of post hoc comparisons; Table S2: Median (25th–75th percentile) residual filling material scores according to final irrigation protocol, NaOCl concentration, and root canal region.

## Abbreviations

The following abbreviations are used in this manuscript:

CLMM	Cumulative link mixed model
LMM	Linear mixed-effects model
CNI	Conventional needle irrigation
CSBS	Calcium silicate-based sealer
EDTA	Ethylenediaminetetraacetic acid
NaOCl	Sodium hypochlorite
PUI	Passive ultrasonic irrigation
SEM	Scanning electron microscopy
SWEEPS	Shock Wave Enhanced Emission Photoacoustic Streaming
XPFR	XP-endo Finisher R
