# Supplementary material for: Effects of Sodium Hypochlorite Concentration and Final Irrigation Protocol on Residual Filling Material in Retreated Oval Root Canals Obturated with a Calcium Silicate-Based Sealer: A Scanning Electron Microscopy Study
Source: Medicina (Kaunas). 2026 Aug 19;62(8):1593. doi: 10.3390/medicina62081593 (PMC13515081; doi:10.3390/medicina62081593)
Supplement: Supplementary file 1 [file medicina-62-01593-s001.zip › medicina-4485884-supplementary.pdf]

Supplementary Table S1. Distribution of residual filling material scores according to final irrigation protocols and results of post hoc comparisons

Final irrigation protocol	Median (25th–75th percentile)
EDDY	3.0 (2.0–3.0)^{A}
CNI	4.0 (4.0–5.0)^{B}
SWEEPS	2.0 (2.0–3.0)^{C}
PUI	3.0 (3.0–3.0)^{AD}
XPFR	3.0 (2.0–3.0)^{AD}

Note: Model results were obtained by averaging over the three NaOCl concentrations and root canal regions. Statistically significant differences among final irrigation protocols within the same column are indicated by uppercase letters (A, B, C, D). Groups indicated by different letters differ significantly according to the Holm-adjusted post hoc test ($p < 0.05$).

Supplementary Table S2. Median (25th–75th percentile) residual filling material scores according to final irrigation protocol, NaOCl concentration, and root canal region.

Final irrigation protocol	NaOCl: 2.5%			NaOCl: 5.25%			NaOCl: 8.25%		
	Coronal	Middle	Apical	Coronal	Middle	Apical	Coronal	Middle	Apical
EDDY	2.5 (2–3)	3 (2.75–3)	3 (3–3.25)	3 (2–3)	3 (2.75–3)	3 (3–3.25)	2 (2–3)	3 (2–3)	3 (3–3)
CNI	4.5 (4–5)	5 (5–5)	5 (5–5)	4 (4–4)	5 (4–5)	5 (4.75–5)	4 (3.75–4)	4 (4–4.25)	4 (4–5)
SWEEPS	2 (2–3)	2 (2–3)	2.5 (2–3)	2 (2–2.25)	2 (2–3)	3 (2.75–3)	2 (2–2)	2 (1–2)	2.5 (2–3)
PUI	3 (2.75–3.25)	3 (2–3)	4 (3–4)	3 (2.75–3)	3 (2–3)	3.5 (3–4)	3 (2.75–3)	3 (2–3)	3 (3–4)
XPFR	3 (2.75–3)	3 (2.75–4)	3 (3–3)	3 (3–3)	3 (2.75–3)	3 (2.75–3)	3 (2–3)	3 (2.75–3)	2.5 (2–3)

Note: Data are presented as median (25th–75th percentile).
